# Novel compound heterozygous mutations in *AMN* cause Imerslund-Gräsbeck syndrome in two half-sisters: a case report

**DOI:** 10.1186/s12881-015-0181-2

**Published:** 2015-06-04

**Authors:** Emma Montgomery, John A. Sayer, Laura A. Baines, Ann Marie Hynes, Virginia Vega-Warner, Sally Johnson, Judith A. Goodship, Edgar A. Otto

**Affiliations:** Renal Services Centre, Freeman Hospital, Newcastle upon Tyne NHS Hospitals Foundation Trust Newcastle upon Tyne, Newcastle upon Tyne, NE7 7DN UK; Institute of Genetic Medicine, Centre for Life, Newcastle Upon Tyne, UK; Department of Pediatrics, University of Michigan, Ann Arbor, MI USA; Paediatric Nephrology, Royal Victoria Infirmary, Newcastle upon Tyne NHS Hospitals Foundation Trust, Newcastle upon Tyne, N1 4LP UK

**Keywords:** Amnionless, Cobalamin deficiency, Anemia, Proteinuria, Vitamin B12, Mutation screening

## Abstract

**Background:**

Imerslund-Gräsbeck Syndrome (IGS) is a rare autosomal recessive disease characterized by intestinal vitamin B12 malabsorption. Clinical features include megaloblastic anemia, recurrent infections, failure to thrive, and proteinuria. Recessive mutations in cubilin (*CUBN*) and in amnionless (*AMN)* have been shown to cause IGS. To date, there are only about 300 cases described worldwide with only 37 different mutations found in *CUBN* and 30 different in the *AMN* gene.

**Case presentation:**

We collected pedigree structure, clinical data, and DNA samples from 2 Caucasian English half-sisters with IGS. Molecular diagnostics was performed by direct Sanger sequencing of all 62 exons of the *CUBN* gene and 12 exons of the *AMN* gene. Because of lack of parental DNA, cloning, and sequencing of multiple plasmid clones was performed to assess the allele of identified mutations. Genetic characterization revealed 2 novel compound heterozygous *AMN* mutations in both half-sisters with IGS. *Trans-*configuration of the mutations was confirmed.

**Conclusion:**

We have identified novel compound heterozygous mutations in *AMN* in a family from the United Kingdom with clinical features of Imerslund-Gräsbeck Syndrome.

## Background

Imerslund-Gräsbeck Syndrome (IGS) is a rare autosomal recessive disorder characterized by intestinal cobalamin (vitamin B12) malabsorption usually leading to megaloblastic anemia in childhood. IGS was first described in 1960 by two physicians, Olga Imerslund from Norway [1] and Ralph Gräsbeck from Finland [2]. Clinical features of IGS include failure to thrive, recurrent infections, anemia, and mild proteinuria. IGS should also be considered in individuals with intermittent nephrotic-range proteinuria [3].

Genetic investigations in affected IGS families from Finland identified bi-allelic mutations in the gene *CUBN*, encoding cubilin [4] whilst in Norwegian families mutations in *AMN*, encoding amnionless were found [5]. Since then, many additional genetically confirmed cases have been reported with about 300 IGS cases published worldwide [6]. IGS is thought to have a prevalence of about 1/200,000 with a geographical concentration in Scandinavia and the Middle East [7]. Most of the published cases have been identified in Finland, Norway and eastern Mediterranean countries frequently in consanguineous families [8]. To date, only 37 different mutations in *CUBN* and 30 different mutations in *AMN* have been reported in the “Human Gene Mutation Database” (HGMD) worldwide (http://www.hgmd.cf.ac.uk/ac/index.php).

Cubilin and amnionless are required for intestinal absorption of intrinsic factor-vitamin B12 complex and renal proximal tubular absorption of filtered plasma proteins. Cubilin is a large (460 kDa) membrane-associated multiligand receptor and amnionless is a 50 kDa type 1 transmembrane protein [9]. Together they function as a heterodimeric complex known as cubam [10]. Both proteins are highly expressed in the small intestine and proximal tubule of the kidney. Within the proximal tubule they interact with megalin (a multi-ligand binding receptor encoded by *LRP2*), allowing reabsorption of low molecular weight (LMW) proteins including albumin [11]. Mutations in *GIF*, encoding gastric intrinsic factor may give a similar hematological and clinical picture, but this is classed as a hereditary intrinsic factor deficiency and should not be confused with IGS [12].

The uptake of LMW proteins within in the kidney initially involves filtration in the glomerulus and then reabsorption within the tubules [13]. Almost all the filtered plasma proteins are reabsorbed in the renal proximal tubule by receptor-mediated endocytosis. The only identified receptors mediating this endocytosis are megalin and cubilin. There are multiple recognised ligands for cubilin and megalin [14]. Once the ligand binds to the receptor at the apical plasma membrane, it is internalised via a vesicle. Acidification within the intra-vesicular lumen then causes the receptor and ligand to separate. The ligand is then transported to its required location and the receptor recycled back into the luminal membrane. Studies in murine models indicate that within the tubule, megalin functions mainly to facilitate the internalisation of the cubilin–albumin complex [15]. As the normal functioning of cubulin is dependent on amnionless, defects in either cubilin or amnionless result in reduced protein reabsorption and subsequent LMW proteinuria. In IGS, published cases have suggested that the proteinuria is non-progressive and has not been associated with clinical kidney disease hence renal function is preserved [16]. Although vitamin B12 replacement corrects haematological parameters, proteinuria usually persists.

Interestingly there have been cases of confirmed IGS, where LMW proteinuria is not present. Currently no clear link has been identified to associate specific mutations to clinical correlations, however with increasing identification of mutations, it appears that functional null mutations in the cubilin or amnionless gene are more likely to be associated with proteinuria than those with a missense mutation [17].

Here we report novel compound heterozygous mutations in *AMN* in 2 half-sisters from the United Kingdom.

## Case presentation

**Fig. 1:**
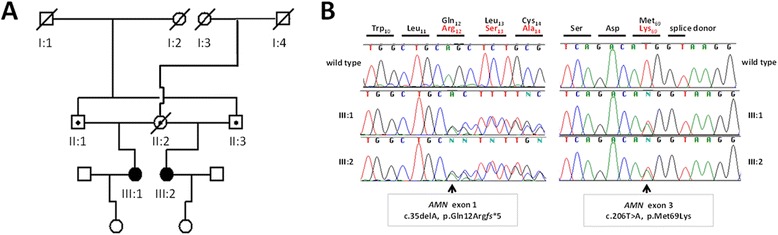
Pedigree diagram of family and chromatograms of *AMN* mutations in a family with Imerslund-Gräsbeck syndrome. **a** Pedigree diagram of a sibship with Imerslund-Gräsbeck syndrome. Circles represent females, squares represent males. Full symbols denote patients with IGS, dots equals presumed carrier status. **b** Chromatograms of 2 novel compound heterozygous mutations in the gene *AMN* (Genbank NM_030943.3), encoding for the protein amnionless, identified in 2 half-sisters (III-1 and III-2) with Imerslund-Gräsbeck syndrome

We identified a Caucasian English family in whom two half-sisters had a clinical diagnosis of IGS (Fig. 1). In both cases, clinical presentation had been at an early age. The eldest sister (III:1) was diagnosed with pernicious anemia at the age of 2 years and was commenced and continued on vitamin B12 supplementation since then. Sub-nephrotic range proteinuria was first identified on urine dipstix testing during her teenage years but was not formally quantified until her first pregnancy at 33 years of age. Her proteinuria was non progressive, and was not associated with hypertension or pre-eclampsia and persisted at similar levels (0.6–0.7 g/24 h) post-natally. Similarly, the younger sister (III:2) also presented with anemia aged 3 years and has been successfully managed with vitamin B12 supplementation. In patient III:2, proteinuria was also first identified at 10 years of age and then quantified (at a sub-nephrotic range) during her pregnancy (age 23 years) and has persisted at similar levels since then. Proteinuria was documented at 0.5 g/24 h at 36 weeks gestation and by delivery (38 weeks gestation) proteinuria had reached 0.9 g/24 h, without the development of systolic hypertension. Postpartum she continued to have persisting sub-nephrotic range proteinuria (0.7 g/24 h). A summary of clinical details is provided in Table 1.

**Table 1:** Clinical features of two half-sisters with Imerslund-Gräsbeck syndrome

ID	Age at diagnosis [years]	B12 deficient	B12 level on treatment [ng/L]	Serum Creatinine [μmol/L]	Urinary protein/creatinine ratio [mg/mmol]	Urinary protein/creatinine ratio [mg/mmol] during pregnancy	Total Vitamin D [nmol/L]	24 h urine protein [g/24 h]	Neurological symptoms
III:1	2	Yes	405	64	50–90	56–84	73	0.6–0.7	No
III:2	3	Yes	743	62	66–71	64–90	76	0.65	No

Both sisters were commenced on 3 monthly vitamin B12 supplementation in early childhood and abnormalities of growth or developmental were not documented. There have been no identified neurological abnormalities in either sibling however multiple urinary tract infections were reported and documented in both sisters throughout their teenage years and into adulthood. The half-sisters continue to receive B12 supplementation however, despite well maintained haemoglobin levels, both siblings continue to experience symptoms of lethargy and tiredness. Sister III:1 became anemic during her pregnancy and required extra B12 supplementation along with iron supplementation.

Careful review of the family history revealed that each of the half-sisters’ fathers were brothers, which was compatible with an autosomal recessive pattern of disease (Fig. 1a). We therefore examined the underlying genetic cause of IGS in this family by performing direct Sanger sequencing of the genes *AMN* (12 exons) and *CUBN* (67 exons) known to be implicated in autosomal recessive IGS. Mutation analysis revealed two novel compound heterozygous mutations in the *AMN* gene in both affected half-sisters. We identified a heterozygous deletion of an adenine nucleotide in exon 1, leading to a frame shift and predicted premature stop 5 codons downstream (c.35delA, p.Gln12Argfs*5) and we found a heterozygous missense mutation (c.206 T > A, p.Met69Lys) in exon 3 of the *AMN* gene (Fig. 1b). Both mutations have not been reported in any public database including “The Exome Aggregation Consortium” (ExAC) data set with exome sequence data obtained from 63,352 individuals (http://exac.broadinstitute.org/).

Since the patients’ mother was deceased and both fathers were uncontactable, no parental DNA material was available for segregation analysis of the mutations. To determine whether the mutations were inherited in *cis* or *trans*, we amplified and cloned the respective genomic region encompassing the location of both identified *AMN* mutations. We successfully amplified and cloned a 1.533 kb PCR product, which included *AMN* exon 1 to 3. Subsequently, we performed direct Sanger sequencing of 24 independent clones, 12 for each patient. Sequencing analysis revealed 12 clones which contained only the exon 1 frameshift mutation (p.Gln12Argfs*5) and 11 clones showing exclusively the exon 3 missense mutation (p.Met69Lys) indicating inheritance from both parents and confirming the presence of a compound heterozygous mutations. Whereas the frameshift mutation is obviously most likely pathogenic, the missense mutation is predicted to be disease causing by various web-based *in silico* prediction programs including “Mutation Taster” (http://www.mutationtaster.org/), ‘Sorting Tolerant From Intolerant’ (SIFT) algorithm (http://siftdna.org/), and Panther (http://www.pantherdb.org/). In contrast, the PolyPhen-2 (http://genetics.bwh.harvard.edu/pph2/index.shtml) program predicts that the amnionless p.Met69Lys missense variant might be a benign amino acid change (score: 0.131).

Additionally, the direct sequencing of the *CUBN* gene revealed the presence of a heterozygous missense variant p.Val2865Met in exon 52 in both affected half-sisters. This variant (rs146847375) has been reported in the Exome Variant Server (EVS) database (http://evs.gs.washington.edu/EVS/) and is present in 6/4294 (0.14 %) European Americans with a pathogenic polyphen-2 prediction score of 0.996. We did not find any additional potential heterozygous variants in *CUBN* in our patients. This almost excludes *CUBN* defects as disease causing in this family, although, we can’t fully exclude the possibility that this heterozygous *CUBN* variant might contribute to the disease phenotype in our patients with bi-allelic *AMN* mutations. *CUBN* is a very large exon-rich gene with more than 200 different missense or truncating variants reported in the EVS database predicted to be possibly or probably protein damaging. Therefore, we reason that the p.Val2865Met missense variant has been an incidental finding. Cases of digenic mutations, where there is one mutant allele in *CUBN* and one in *AMN* have to our knowledge never been reported.

## Conclusion

Using targeted Sanger sequencing of *AMN* and *CUBN*, we identified novel compound heterozygous mutations in *AMN* in a family from the United Kingdom with typical clinical features of Imerslund-Gräsbeck Syndrome.

## Consent

Following informed consent, DNA was obtained from both affected patients. This study was approved by the Northern and Yorkshire Regional Ethics Committee. Patients have given their consent for the Case reports to be published.

## Abbreviations

EVS: Exome Variant Server
ExAC: Exome Aggregation Consortium
HGMD: Human Gene Mutation Database
IGS: Imerslund-Gräsbeck Syndrome
LMW: Low molecular weight
SIFT: Sorting Tolerant From Intolerant
